# Fine-needle aspiration to improve diagnosis of melioidosis of the head and neck in children: a study from Sarawak, Malaysia

**DOI:** 10.1186/s12879-021-06754-9

**Published:** 2021-10-15

**Authors:** Anand Mohan, Yuwana Podin, Da-Wei Liew, Jeevithaa Mahendra Kumar, Peter Sie-Teck Lau, Yee-Yen Tan, Yi-Pinn Tai, Ranveer Singh Gill, Ram Shanmugam, Su-Lin Chien, Lee-See Tan, Nurul Asiah Mat Sani, Kamilah Manan, Mong-How Ooi

**Affiliations:** 1 Department of Pediatrics, Bintulu Hospital, Ministry of Health Malaysia, Bintulu, Sarawak Malaysia; 2 Institute of Health and Community Medicine, Universiti Malaysia Sarawak, Kota Samarahan, Sarawak Malaysia; 3 Department of Otorhinolaryngology, Bintulu Hospital, Ministry of Health Malaysia, Bintulu, Sarawak Malaysia; 4 Department of Pathology, Bintulu Hospital, Ministry of Health Malaysia, Bintulu, Sarawak Malaysia; 5 Department of Radiology, Bintulu Hospital, Ministry of Health Malaysia, Bintulu, Sarawak Malaysia; 6 Department of Pediatrics, Sarawak General Hospital, Ministry of Health Malaysia, Kuching, Sarawak Malaysia

**Keywords:** Melioidosis, Children, Head and neck, Lymph nodes, Diagnosis, Malaysia

## Abstract

**Background:**

Melioidosis, the infection caused by *Burkholderia pseudomallei*, is associated with a high case fatality rate, due in part to difficulties in clinical recognition and diagnostic confirmation of the disease. Although head and neck involvement is common in children, specific disease manifestations differ between geographic regions. The aim of this study was to provide a detailed description of melioidosis of the head and neck among children in Sarawak, Malaysia, and determine if fine-needle aspiration of suspected head or neck lesions could improve melioidosis diagnosis.

**Methods:**

We conducted a retrospective descriptive study of all children aged < 12 years with culture-confirmed melioidosis presenting with head and neck manifestations and admitted to Bintulu Hospital in Sarawak, Malaysia, from January 2011 until December 2020. Fine-needle aspiration of head and neck lesions suspected to be due to melioidosis with inoculation in blood culture bottles (FNA + BCB) was used from the beginning of 2016.

**Results:**

Of 34 children with culture-confirmed melioidosis, 20 (59%) had an infection involving one or more sites in the head and neck. Of these, 17 (85%) were diagnosed in or after 2016. Cervical lymph nodes were the most common organ or site affected, involved in 19 (95%) children. Clinical presentations of *B. pseudomallei* lymph node infections were highly variable. Five (25%) children had salivary gland involvement. Lacrimal gland involvement (dacryocystitis) and skin or soft tissue infection (scalp abscess) were less frequent. *B. pseudomallei* was isolated from the head or neck using FNA + BCB in 15 (75%) children and by standard culture methods of direct plating of pus on agar following incision and drainage in only 2 (10%) children. *B. pseudomallei* was isolated from non-head or neck specimens or blood in 3 (15%) children.

**Conclusions:**

Manifestations of pediatric head and neck melioidosis in Sarawak, Malaysia, differ from those of other regions. Fine-needle aspiration, mainly of affected cervical lymph nodes, facilitates *B. pseudomallei* detection and enables confirmation of melioidosis infections.

**Supplementary Information:**

The online version contains supplementary material available at 10.1186/s12879-021-06754-9.

## Background

Melioidosis, the infection caused by the environmental saprophyte *Burkholderia pseudomallei*, is associated with a high case fatality rate [1, 2]. This is due partly to the extensive intrinsic antibiotic resistance of the organism [3], but also to difficulties in case recognition and diagnosis [4, 5], especially in rural settings where the disease commonly occurs [6, 7].

Difficulties in clinical recognition of melioidosis arise mainly due to the varied and non-specific presentation of patients. Patients from different regions can have different disease manifestations [8], and there are also variations between pediatric and adult presentations of the disease [9, 10]. Difficulties in confirming suspected infections further lengthen the diagnostic process. Laboratory investigations, including bacterial culture, rapid antigen detection, direct real-time polymerase chain reaction assays (PCR), and serology all suffer from limited sensitivity or specificity [8, 11, 12]. For example, sensitivity and specificity of PCR on clinical isolates were reported to be 73.2% and 89.2%, respectively [13]. Bacterial culture, although 100% specific, may have a sensitivity of only 60% [11].

In view of and despite the limitations of the various diagnostic modalities, adequate sampling of the site of infection is key in diagnosing melioidosis [14]. The use of fine-needle aspiration to obtain pus and tissue samples from various body sites, including mediastinal lymph nodes, spleen, and prostate, has been reported to have enabled confirmation of melioidosis infections [15–17]. This minimally invasive technique may be particularly useful for difficult to access sites and when surgical biopsies are not possible or available [18, 19].

Bintulu Hospital is a 302-bed hospital which provides primary and secondary health services to a total population of 256,000, including 72,000 children aged < 15 years, residing in Bintulu Division and Belaga district of Kapit Division in central Sarawak, Malaysian Borneo. Although adult general surgery and otorhinolaryngology services are available, specialized pediatric services are only available in a tertiary referral center located over 300 km away in southern Sarawak.

In 2017, we reported a broad description of melioidosis infections among a total of 42 children presenting to three hospitals in central Sarawak, including Bintulu Hospital [20]. One interesting finding was that cervical lymph node infections were frequently present, documented in 26% of cases. We also found that 24% of all children had a fatal outcome. In comparison, the pediatric case fatality rate was lower in other regions [9, 21]. The higher apparent fatality rate found in Sarawak may in fact suggest that many children with localized or less severe melioidosis had not been diagnosed, possibly related to the difficulties with diagnosis and limited resources. The aim of this study was to provide a detailed description of head and neck melioidosis among children in Sarawak and determine whether fine-needle aspiration of suspected head and neck lesions could facilitate the detection of *B. pseudomallei* and improve melioidosis diagnosis.

## Methods

We performed a retrospective descriptive study of all children with melioidosis of the head and neck presenting to Bintulu Hospital in Sarawak, Malaysian Borneo, over a 10-year period from January 2011 to December 2020.

A child was classified as having melioidosis of the head and neck if any structure or organ in the head or neck, with the exception of the nervous system organs, were involved (based on abnormal physical and/or radiological findings) at presentation in a culture-confirmed melioidosis case. Culture-confirmed melioidosis was defined by isolation of *B. pseudomallei* from any clinical sample, including from non-head or neck organ-sites. To identify cases of melioidosis of the head and neck, we first conducted a manual search of the microbiology laboratory logbooks and electronic database to identify children aged < 12 years with culture-confirmed melioidosis. Medical records were then retrieved and reviewed to identify those who had melioidosis of the head and neck. Details on demography, clinical, laboratory, and radiological findings, case management, and outcome were collected using a standardized case report form.

Based on admission body weight, poor nutritional status was diagnosed in children aged ≤ 10 years if weight-for-age was below − 2 z-score using the World Health Organization Child Growth Standards [22], while children aged > 10 years were considered to have poor nutritional status if their body weight was below the 3rd percentile using the Centers for Disease Control and Prevention weight-for-age percentiles [23]. Lymphadenopathy was defined as an enlarged, non-tender lymph node with no obvious signs of inflammation. Lymphadenitis was defined as an enlarged, tender lymph node, with the presence of signs of inflammation. A lymph node abscess was diagnosed based on the presence of fluctuance in an enlarged lymph node. Melioidosis was considered to be localized if a single or multiple organ-site(s) of involvement were confined to the head and neck, in the absence of a positive blood culture and clinical/radiological/microbiological evidence of dissemination to other non-head or neck sites. Disseminated melioidosis was defined as the presence of infection in ≥ 1 discrete body sites other than those found in the head and neck and/or a positive blood culture. Septic shock was defined by the presence of hypotension (systolic blood pressure below the 5th percentile) with evidence of inadequate tissue perfusion unresponsive to fluid replacement [24]. Leukocytosis was defined by an elevated white blood cell count > 14 × 10^9^/L in children < 2 years, > 12 × 10^9^/L in children 2–9 years, and > 10.5 × 10^9^/L in children > 9 years [25]. Neutrophilia was defined as an absolute neutrophil count > 8.5 × 10^9^/L in children < 6 years and > 8.0 × 10^9^/L in children 6 years or older. Monocytosis was defined as an absolute monocyte count > 1.0 × 10^9^/L. Thrombocytosis was defined as a platelet count > 400 × 10^9^/L. A melioidosis-active antibiotic regimen was defined as the use of either ceftazidime or a carbapenem during the intensive phase and oral trimethoprim–sulfamethoxazole during the eradication phase, with amoxicillin–clavulanate or doxycycline used as second-line oral agents when resistance, contraindications, or intolerance to trimethoprim–sulfamethoxazole was present [13].

Before 2016, pus and other samples from the head and neck were mainly collected during incision and drainage procedures using sterile bottles before being transported as quickly as possible to the microbiology laboratory. These specimens were then cultured directly on blood agar (BA), chocolate agar (CA), and MacConkey agar (MAC) (standard methods). From 2016, in an attempt to improve culture-confirmation of melioidosis infections, a fine-needle aspiration technique with inoculation in blood culture bottles (FNA + BCB) was used in addition to standard methods. With this method, fine-needle aspiration of affected lymph nodes and other lesions in the head and neck were performed using a 23-gauge needle, similar to fine-needle aspiration biopsy procedures previously described [26]. Aspirated contents were inoculated directly into pediatric BACTEC blood culture bottles (Becton Dickinson, USA). When minimal (i.e., pus/tissue present only in the hub of the needle) or no pus/tissue was aspirated, a small amount of culture medium from the blood culture bottle was aspirated through the needle into the syringe and flushed back into the bottle. Culture bottles were then incubated in the BACTEC blood culture system (Becton Dickinson, USA). Positive growth was subcultured onto BA, CA, and MAC.

*B. pseudomallei* was identified with either API^®^20NE (BioMérieux, France) or BBL™ Crystal™ Identification Systems (Becton Dickinson, USA). Blood samples collected from patients were subjected to the BACTEC blood culture system.

Statistical analysis was performed using SPSS Statistics 21. Continuous variables were presented using the median and interquartile range (IQR).

## Results

### Patient demographics and clinical manifestations

Thirty-four children had culture-confirmed melioidosis during the 10-year study period. Medical records of all cases were available for review. Head or neck manifestations were present at admission in 20 (59%) children. Of these, only 3 (15%) were diagnosed between 2011 and 2015, while 17 (85%) were diagnosed between 2016 and 2020.

The median age of children with head or neck melioidosis was 8.0 years (IQR 2.0–9.7 years, range 1.0–10.9 years) (Table 1). The median duration of symptoms prior to admission was 14 days (IQR 8–30 days, range 4–42 days). Fever was present in most cases (n = 17). The head or neck manifestation was the primary reason for admission in all but two children. In the remaining 2, head or neck lesions were detected at physical examination following presentations with non-head or neck manifestations (pneumonia and septic arthritis in one, pyrexia of unknown origin in another).

**Table 1:** Summary of the clinico-epidemiological characteristics of 20 children with head and neck melioidosis admitted to Bintulu Hospital (Sarawak, Malaysia) between 2011 and 2020

Variable	Number (%)
Male gender	9 (45)
Age, years, median (IQR)	8.0 (2.0–9.7)
Pre-existing medical condition	0 (0)
Poor nutritional status	7 (35)
Time between onset and admission, days, median (IQR)	14 (8–30)
History of fever or fever on admission	17 (85)
Site(s) of infection in the head and neck^a^	
Cervical lymph node	19 (95)
Salivary gland	5 (25)
Lacrimal gland	2 (10)
Skin/soft tissue	1 (5)
Liver and/or spleen abscess(es) present^b^	11 (58)
Bacteremia	2 (10)
Disseminated disease	13 (65)
Septic shock	1 (5)
Respiratory failure	2 (10)
Required surgical drainage procedure	7 (35)
Death	1 (5)

Data are number (%) unless otherwise indicated

*IQR* interquartile range

^a^Some children had more than 1 site of infection in the head and neck

^b^In the 19 children who had abdominal ultrasound examination

Cervical lymph node infection was the most common manifestation of head or neck melioidosis, present in 19 of the 20 children (Table 1). Among 18 who had symptoms/signs of cervical lymph node involvement, presentations were highly variable and included lymphadenitis (n = 8), lymphadenopathy (n = 6), and lymph node abscesses (n = 4) (Table 2). Jugular or posterior triangle lymph node involvement was recorded in 12 (67%) cases, while submandibular and submental involvement was recorded in 5 (28%) and 1 (6%) cases, respectively. No pre-auricular, post-auricular, occipital, or supraclavicular lymph node involvement was recorded. Multiple nodes were affected in 11 (61%) children. Nine (50%) had bilateral involvement. The largest node in each individual measured between 1.5 and 6 cm. In 1 child, enlarged cervical lymph nodes measuring up to 1.6 cm were only detected on computed tomography during investigation for bilateral parotid gland abscesses.

**Table 2:** Clinical and biological characteristics of 20 children with head and neck melioidosis admitted to Bintulu Hospital (Sarawak, Malaysia) between 2011 and 2020

No	Year of admission	Age-group, years	Presenting manifestation	Method used for bacterial culture of head or neck lesion		Blood culture
				FNA + BCB	Standard	
1	2011	2–5	Cervical lymphadenopathy, left	ND	B.p	Negative
2	2011	< 2	Scalp abscess	ND	B.p	Cont
3	2014	> 5	Cervical lymphadenitis, bilateral	ND	ND	B.p
4	2016	< 2	Cervical lymphadenitis, bilateral	B.p	No/min. asp	Negative
5	2016	< 2	Cervical lymphadenitis, left	B.p	No/min. asp	Negative
6	2017	> 5	Cervical lymph node abscess, right	B.p	Negative	Negative
7	2017	> 5	Cervical lymphadenopathy, left	B.p	ND	Negative
8	2018	> 5	Cervical lymphadenitis, bilateral	B.p	B.p	Negative
9	2018	> 5	Cervical lymphadenitis, bilateral^a^	ND	ND	B.p
10	2018	2–5	Cervical lymph node abscess, left	B.p	Negative	Negative
11	2018	> 5	Cervical lymphadenitis, bilateral	B.p	Negative	Negative
12	2018	< 2	Cervical lymphadenopathy, left^a^	B.p	ND	Negative
13	2018	> 5	Cervical lymphadenopathy, bilateral	B.p	Negative	Negative
14	2019	> 5	Cervical lymphadenitis, right	B.p	No/min. asp	Negative
15	2019	> 5	Cervical lymphadenitis, bilateral	B.p	No/min. asp	Negative
16	2019	> 5	Cervical lymphadenopathy, bilateral	ND	ND	Negative^b^
17	2020	> 5	Parotid abscess, bilateral	B.p	Negative	Negative
18	2020	2–5	Cervical lymph node abscess, bilateral	B.p	ND	Negative
19	2020	> 5	Cervical lymphadenopathy, right	B.p	No/min. asp	Negative
20	2020	< 2	Cervical lymph node abscess, left	B.p	Negative	Negative

FNA + BCB, Fine-needle aspiration with inoculation in a blood culture bottle; ND, not done; B.p, *Burkholderia pseudomallei*; Cont, contaminated; No/min. asp, No or minimal pus/fluid aspirated

^a^Head and neck lesions were not the presenting manifestation but were found during physical examination at admission. Case 9 presented with pneumonia and septic arthritis of the right ankle and left knee while Case 12 presented with pyrexia of unknown origin

^b^*Burkholderia pseudomallei* was cultured from a soft tissue abscess on the left foot

Salivary gland involvement was present in 5 (25%) children. However, only 1 presented with symptoms/signs of salivary gland disease (bilateral parotid abscesses). In the remainder, the salivary gland involvement was detected on ultrasound examination, performed in children who had presented with cervical lymph node infection. Ultrasound findings in these four children showed a unilateral parotid abscess measuring between 2 and 3 cm (n = 2), multiple bilateral parotid abscesses smaller than 0.5 cm (n = 1), and unilateral enlarged submandibular gland with intra-glandular lymph nodes (n = 1), in addition to the enlarged cervical lymph nodes. Overall, 67% (12/18) of children who presented with cervical lymph node manifestations had head and neck ultrasonography; 33% (4/12) were found to have occult salivary gland involvement.

**Fig. 1:**
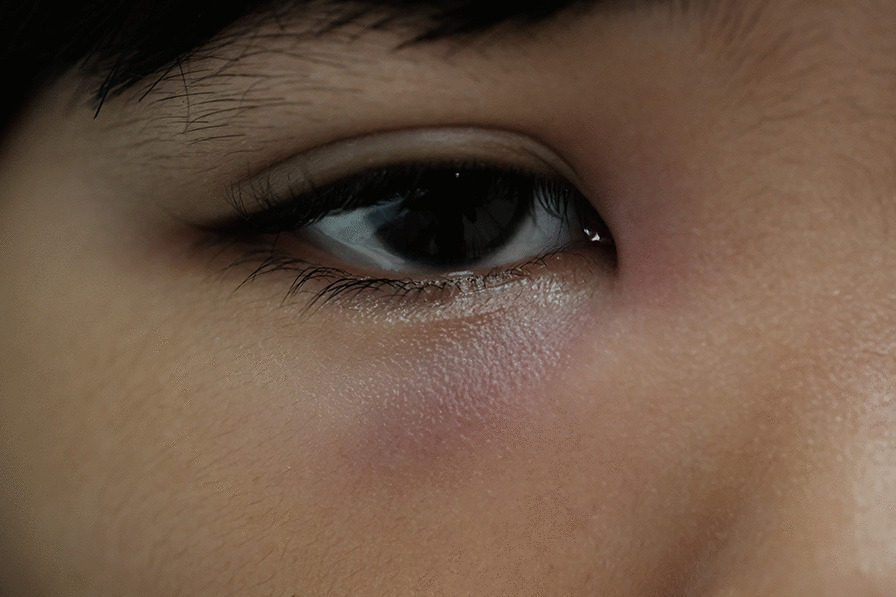
Image showing erythema and swelling of the right lower eyelid in a 9-year 3-month old girl with unilateral dacryocystitis due to melioidosis admitted to Bintulu Hospital (Sarawak, Malaysia) in 2019

Two children had lacrimal gland involvement. Both presented with cervical lymph node infection: one had bilateral lymphadenopathy and was found to have concomitant unilateral dacryocystitis; the other had unilateral lymphadenitis with ipsilateral dacryocystitis (Fig. 1). Skin or soft tissue infection of the head or neck was present in only one child. This was a 1-year 8-month old girl who presented with a 4 cm occipital scalp abscess.

Of the 20 children with head and neck melioidosis, 7 (35%) had a localized infection, whereas 13 (65%) had disseminated disease. In the 13 children with disseminated melioidosis, non-head or neck sites of infection included spleen (n = 11), lungs (n = 2), joints (n = 2), non-head or neck soft tissue (n = 2), axillary lymph node (n = 1), and brain (n = 1).

### Laboratory findings

Leukocytosis was present in 13 (65%) of the 20 children. Of 16 (80%) with differential counts, 13 (81%) had neutrophilia and 10 (63%) had monocytosis. Lymphocyte counts were normal in all but two children. The median hemoglobin was 11.0 g/dL (IQR 10.5–12.2 g/dL). Thrombocytosis was present in 13 (65%) patients. The median erythrocyte sedimentary rate was 89 mm/h (IQR 70–105 mm/h).

Seven (39%) of the 18 children who presented with cervical lymph node infection had histologic examination. Five (71%) were reported to have epithelioid granulomas; among these, multinucleated giant cells and caseous necrosis were found in 4 and 1, respectively. Histology of the cervical lymph nodes in the remaining two cases showed neutrophils and lymphocytes in one, and sparsely cellular smears with poorly preserved histiocytes in the other.

### Detection of *B. pseudomallei* in melioidosis head and neck infections

The source of positive isolates in the 20 cases included pus/tissue from cervical lymph nodes (n = 15), salivary gland (n = 1), head or neck skin/soft tissue abscess (n = 1), and non-head or neck skin/soft tissue abscess (n = 1). *B. pseudomallei* was isolated from blood in the two remaining cases.

Of the three cases of melioidosis of the head and neck identified between 2011 and 2015 (before the initiation of FNA + BCB), 2 were diagnosed by isolation of *B. pseudomallei* from the head or neck (i.e., using standard methods of direct plating of pus/tissue following incision and drainage) (Table 2). Between 2016 and 2020 (after initiation of FNA + BCB), 15 of the 17 cases of melioidosis of the head and neck identified were diagnosed by isolation of *B. pseudomallei* from the head or neck. In all 15, *B. pseudomallei* was detected using FNA + BCB. In contrast, with standard methods of direct plating of pus/tissue obtained from the fine-needle aspiration procedure, *B. pseudomallei* was isolated in only 1 of these 15 children (Additional file 1: Figure S1). The remaining 14 had either negative standard method cultures (n = 6), no or minimal aspirates rendering standard methods impossible (n = 5), or no standard method performed (n = 3). In 9 (60%) of 15, *B. pseudomallei* was isolated even though FNA + BCB was performed after initiation of melioidosis-active antibiotics (Additional file 2: Table S1). In these nine children, the median duration of antibiotics prior to FNA + BCB was 2 days (range 1–28 days). In 12 (86%) of 14 patients with data available, the presence of Gram-negative bacilli in the FNA + BCB specimen was reported within 2 days of the procedure. The median duration between FNA + BCB procedure and *B. pseudomallei* identification was 4 days (IQR 4–5 days). No significant difference was noted in the duration between FNA + BCB procedure and *B. pseudomallei* identification for cases with no/minimal aspirate (and no standard method performed) and those with more substantial volume of pus/tissue aspirate (as indicated by the availability of pus/tissue for standard culture methods) (*P* = 0.8).

All children had blood cultures performed. Of the 18 without bacteremia, 7 (39%) had multiple blood cultures performed.

### Case management and outcome

One (5%) child had a fatal outcome. This was a 7-year 10-month old boy who presented in 2014 with bilateral cervical lymphadenitis, disseminated sites of infection (lungs, brain), bacteremia, respiratory failure, and septic shock on admission.

Twelve (60%) children were initially treated empirically for pyogenic infections other than melioidosis; antibiotics used included ampicillin, cloxacillin, cefuroxime, ampicillin/sulbactam, and metronidazole. Four (20%) were discharged home to continue oral antibiotics but returned with unresolved symptoms.

All ultimately received melioidosis-active antibiotic regimens; surgical incision and drainage was required in 7 (35%) cases. Among survivors, the median duration of parenteral intensive phase treatment was 21 days (range 13–45 days). All had clinical resolution of the presenting manifestations. For eradication, 9 (47%) children received trimethoprim–sulfamethoxazole, 5 (26%) received amoxicillin–clavulanate, and 5 (26%) others received a combination of amoxicillin–clavulanate and doxycycline. The median duration of oral eradication therapy was 17 weeks (range 12–28 weeks). No relapses were recorded during the study period.

No complications from the fine-needle aspiration procedure were recorded.

## Discussion

This study reports the demography, clinical presentations, laboratory findings, treatment, and outcome of children with melioidosis of the head and neck in Sarawak, Malaysian Borneo. We show that children with head or neck melioidosis in Sarawak have distinct manifestations with involvement predominantly of cervical lymph nodes. In addition, we describe how the use of fine-needle aspiration improves diagnosis of melioidosis in these children.

The head and neck region was a major site of melioidosis infection in Sarawakian children, with melioidosis of the head and neck representing nearly 60% of the melioidosis cases diagnosed between 2011 and 2020. Head and neck infections are a well described feature of pediatric melioidosis; in Cambodia and Thailand, 65% and 33% of children, respectively, are reported to have head or neck involvement [27, 28]. Interestingly, however, head and neck infections are rarely reported in children in northern Australia [6, 9]. In adults, head and neck melioidosis is uncommon [29, 30]. The high prevalence of head or neck infections among children in Southeast Asia is thought to be related to acquisition of *B. pseudomallei* through the oral/nasal mucosa or conjunctiva, possibly via exposure to contaminated water sources, as opposed to inhalation and ingestion postulated in adults with pneumonia or bacteraemia and skin inoculation among children in northern Australia [31, 32].

In our study, cervical lymph nodes were the most common organ-site involved in the head and neck, and differentiating *B. pseudomallei* infection from other common causes of cervical lymph node disease, including pyogenic or tuberculous (TB) infection, by clinical, routine laboratory, and even histologic examination was difficult. Cervical lymph node involvement was present in 19 (95%) of our 20 cases and was the primary reason for admission in 80% (16/20) of the cases. These results contrast with findings from Cambodia and Thailand, where salivary glands, and in particular parotid glands, are the most common site of infection [33, 34]. It is unlikely that this discrepancy was due to misdiagnosis of parotitis among children in our study, as none of the children had pre-auricular swelling. Additionally, 2/3 had ultrasound imaging confirming cervical lymph node involvement, although in 25% small parotid abscesses were simultaneously found. It is uncertain whether parotitis is being over-diagnosed in the rest of Southeast Asia, although it remains possible that a true difference in predilection of organ-sites exists. Reasons for this, however, are not yet known. In our study, a broad range of cervical lymph node presentations from unilateral painless adenopathy to bilateral lymph node abscesses were found. Most laboratory findings were non-specific except for monocytosis which occurred in 63%. Whilst monocytosis has been shown to be a useful predictor of TB [35], the utility of this hematological finding for melioidosis diagnosis has not been evaluated. Histological examination showed findings that were either indistinguishable from TB or were non-specific. Thus, a high index of suspicion for melioidosis is required in children presenting with cervical lymph node swelling in melioidosis-endemic regions, and microbiological confirmation is essential.

Fine-needle aspiration, mainly of affected cervical lymph nodes, improved the diagnosis of melioidosis of the head and neck among children in this study, as it facilitated sampling of the site of infection and enabled microbiological confirmation of melioidosis (Additional file 3: Table S2). Indeed, the diagnosis was obtained in 75% of our cases using fine-needle aspiration, and these cases were not diagnosed by any other method. In addition, over 5 times more culture-confirmed melioidosis cases were detected in the second half of the study, mainly attributable to the use of fine-needle aspiration. Before 2016, with the exception of the three cases reported in this study, most diagnoses of head and neck melioidosis in Bintulu Hospital were made based on clinical and serological findings. Blood cultures were usually negative, and unless obvious collections were present, pus/tissue from lesions were rarely sampled as the lack of pediatric surgical/otorhinolaryngology services resulted in a reluctance to perform biopsies/drainage procedures. When drainage procedures were performed (usually late in the course of the illness), pus cultures were frequently negative. In an attempt to narrow the diagnostic gap, FNA + BCB was initiated at the beginning of 2016. Fine-needle aspiration is a simple and safe procedure, which may be performed at the bedside, and has routinely been used in the diagnosis of TB lymphadenitis [36, 37]. Complications of fine-needle aspiration, including bleeding, damage to surrounding structures, fistula creation, or seeding of tissue planes with infection or neoplastic cells, are rare [38]. In contrast, open/excisional biopsies are more invasive and require surgical expertise and general anesthesia. Fine-needle aspiration may thus be a useful option in the resource-limited regions where most melioidosis infections occur.

The use of blood culture bottles for bacterial culture of non-blood specimens has previously been reported. For example, improved yield has been documented in the culture of synovial fluid, pleural fluid, and brain abscess biopsies [39–41]. A number of reasons may explain the higher rates of *B. pseudomallei* isolation with the use of blood culture bottles compared to direct plating on agar reported in the present study. Probably most importantly, the use of blood culture bottles enabled detection of *B. pseudomallei* even when no or minimal pus/tissue was present, using a technique of ‘rinsing’ the biopsy needle and syringe by aspirating contents of the blood culture bottle. This similar technique has been shown to be useful in culture-confirmation of TB lymphadenitis [42]. The ability to isolate *B. pseudomallei* from infected lymph nodes even when no pus is present, with similar detection times as for large samples of purulent aspirates, suggests a sensitive approach with the potential to enable earlier diagnosis. Other reasons for the improved yield may include the benefits of liquid culture media versus solid culture media and the presence of antibiotic-binding resins in commercial blood culture media [43–45]. However, it is probable that any suitable enrichment broth (other than blood culture media) would have performed equally well, and this should be evaluated in future studies.

Even though bacteremia was rarely detected among children with melioidosis of the head and neck, disseminated infections were diagnosed in 65% of cases. The spleen was the most common organ affected in disseminated infections; occult splenic abscesses were present in 85% (11/13) of children with disseminated disease and in 58% (11/19) overall. As previously reported, detection of splenic abscesses by abdominal ultrasonography offers a useful and rapid clue to melioidosis diagnosis [46]. In addition, the detection of occult splenic abscesses in children with seemingly localized head and neck melioidosis in this study is also important in view of recent suggestions that localized infections might adequately be treated with oral antibiotics alone [21]. Clearly, a thorough diagnostic work-up to exclude occult dissemination is needed before such a treatment decision can be considered. Alternatively, in melioidosis-endemic regions, the detection of a head or neck focus of infection (e.g., cervical lymph node infection) in a child presenting primarily with infection in another organ-site, for example pneumonia or septic arthritis, may suggest a diagnosis of melioidosis and the need for empirical melioidosis-active antibiotics.

This study has several limitations. Firstly, throat swabs for culture of *B. pseudomallei* were not performed. Throat swabs have been shown to be useful in the diagnosis of melioidosis and may have been especially beneficial in the present study, as some of the children could have had a pharyngo-cervical syndrome as reported in previous studies [27, 47]. Throat swab culture for *B. pseudomallei* requires the use of selective media, such as Ashdown’s agar. However, Ashdown’s agar cannot be used in Sarawak as the majority of cases are caused by gentamicin-susceptible *B. pseudomallei* [48], while other *B. pseudomallei* selective media and modified Ashdown (with colistin instead of gentamicin) are not readily available. Secondly, this study does not report cases of suspected head and neck melioidosis in which all cultures, including FNA + BCB, were negative and as such cannot inform on sensitivity of the method. This is mainly related to the study methodology and the lack of a gold standard for diagnosis. Another limitation of the study was that no cost-effectiveness calculations were undertaken. These may be important, as melioidosis is largely an affliction of resource-limited regions. Other limitations include the small sample size, the possibility of selection bias related to the monocentric nature of the study, and limitations inherent to the retrospective (bias of information) descriptive (bias of confusion) study design. Despite these limitations, the study manages to provide a fairly comprehensive characterization of pediatric head and neck melioidosis in Sarawak, of which very limited data was previously available.

## Conclusions

Nearly 60% of children with melioidosis in Sarawak had head or neck involvement. Cervical lymph nodes were the most common site of infection in the head and neck; salivary and lacrimal gland infections also occurred but were less frequently observed. Fine-needle aspiration, mainly of affected cervical lymph nodes, was extremely useful in enabling microbiological confirmation of melioidosis, allowing the diagnosis of 75% of our cases. Further studies are needed to confirm the sensitivity, specificity and cost-effectiveness of this simple and safe technique, which may be particularly useful in resource-constrained regions.

## Supplementary Information

**Additional file 1: Figure S1. **Source of positive *B. pseudomallei* isolate and method used to obtain and isolate the organism in 20 children with head and neck melioidosis in Bintulu Hospital (Sarawak, Malaysia) between 2011 and 2020.

**Additional file 2: Table S1.** Detailed characteristics of the head or neck bacterial culture procedure in the 20 children with melioidosis of the head and neck in Bintulu Hospital (Sarawak, Malaysia) between 2011 and 2020.

**Additional file 3: Table S2. **Patient, health personnel, and infrastructure characteristics present in Bintulu Hospital (Sarawak, Malaysia) related to the improved diagnosis of melioidosis of the head and neck in children with the use of fine-needle aspiration versus surgical biopsy.

## Data Availability

The datasets used and analyzed during the current study are available from the corresponding author on reasonable request.

## Abbreviations

BA: Blood agar
CA: Chocolate agar
MAC: MacConkey agar
FNA + BCB: Fine-needle aspiration with inoculation in a blood culture bottle
IQR: Interquartile range
TB: Tuberculosis
