# Supplementary material for: Fine-needle aspiration to improve diagnosis of melioidosis of the head and neck in children: a study from Sarawak, Malaysia
Source: BMC Infect Dis. 2021 Oct 15;21:1069. doi: 10.1186/s12879-021-06754-9 (PMC8520244; doi:10.1186/s12879-021-06754-9)
Supplement: Supplementary file 1 — Additional file 1: Figure S1. Source of positive B. pseudomallei isolate and method used to obtain and isolate the organism in 20 children with head and neck melioidosis in Bintulu Hospital (Sarawak, Malaysia) between 2011 and 2020. [file 12879_2021_6754_MOESM1_ESM.docx]

**2016-2020**

**(After initiation of FNA+BCB)**

**n=17**

**2011-2015**

**(Before use of FNA+BCB)**

**n=3**

**Period of study**

**Non- head and neck**

**n=2**

**Head and neck**

**n=15**

**Head and neck**

**n=2**

**Source of positive**

***B. pseudomallei* isolate**

**Non- head and neck**

**n=1**

**Incision and drainage with standard methods**

**n=2**

**FNA+BCB**

**n=15**

**Method used to obtain and**

**isolate *B. pseudomallei***

**Positive**

**n=1**

**Results of standard method cultures**

**i.e., direct plating of pus/tissue**

**obtained during fine-needle aspiration**

**No/minimal aspirate**

**n=5**

**Not attempted**

**n=3**

**Negative**

**n=6**
