# Supplementary material for: Fine-needle aspiration to improve diagnosis of melioidosis of the head and neck in children: a study from Sarawak, Malaysia
Source: BMC Infect Dis. 2021 Oct 15;21:1069. doi: 10.1186/s12879-021-06754-9 (PMC8520244; doi:10.1186/s12879-021-06754-9)
Supplement: Supplementary file 2 — Additional file 2: Table S1. Detailed characteristics of the head or neck bacterial culture procedure in the 20 children with melioidosis of the head and neck in Bintulu Hospital (Sarawak, Malaysia) between 2011 and 2020. [file 12879_2021_6754_MOESM2_ESM.docx]

**Table S1**

**Detailed characteristics of the head or neck bacterial culture procedure in the 20 children with melioidosis of the head and neck in Bintulu Hospital (Sarawak, Malaysia) between 2011 and 2020**

| No | Year of admission | Method used for bacterial culture of head or neck lesion | | Duration between admission and procedure (days) | Number of days on melioidosis-active antibiotics before procedure performed | Duration between procedure and report (days) | |
| --- | --- | --- | --- | --- | --- | --- | --- |
|  |  | FNA+BCB | Standard |  |  | Gram stain | Full report |
| 1 | 2011 | ND | B.p | 12 | Before antibiotics | 1 | 2 |
| 2 | 2011 | ND | B.p | 1 | Before antibiotics | NR | 3 |
| 3 | 2014 | ND | ND | - | - | 2* | 3* |
| 4 | 2016 | B.p | No/min. asp | 3 | 1 | NR | 4 |
| 5 | 2016 | B.p | No/min. asp | 2 | Before antibiotics | 2 | 5 |
| 6 | 2017 | B.p | Negative | 1 | Before antibiotics | 2 | 5 |
| 7 | 2017 | B.p | ND | 2 | Before antibiotics | 2 | 5 |
| 8 | 2018 | B.p | B.p | 2 | 1 | 2, NR^†^ | 4, 4^†^ |
| 9 | 2018 | ND | ND | - | - | 3* | 6* |
| 10 | 2018 | B.p | Negative | 2 | Before antibiotics | 2 | 4 |
| 11 | 2018 | B.p | Negative | 2 | 2 | 3 | 5 |
| 12 | 2018 | B.p | ND | 30 | 28 | 3 | 6 |
| 13 | 2018 | B.p | Negative | 2 | 2 | 2 | 4 |
| 14 | 2019 | B.p | No/min. asp | 5 | 5 | 1 | 3 |
| 15 | 2019 | B.p | No/min. asp | 10 | 10 | 2 | 4 |
| 16 | 2019 | ND | ND | - | - | 1* | 3* |
| 17 | 2020 | B.p | Negative | 0 | Before antibiotics | 2 | 4 |
| 18 | 2020 | B.p | ND | 0 | Before antibiotics | 2 | 4 |
| 19 | 2020 | B.p | No/min. asp | 2 | 2 | 2 | 5 |
| 20 | 2020 | B.p | Negative | 4 | 4 | 2 | 3 |

FNA+BCB, Fine-needle aspiration with inoculation in a blood culture bottle; ND, not done; B.p, *Burkholderia pseudomallei*; NR, data not recorded; No/min. asp, No or minimal pus/fluid aspirated.

*Represents time to report of culture of non-head or neck specimen.

^†^Duration between procedure and report for standard culture method.
