# Supplementary material for: Fine-needle aspiration to improve diagnosis of melioidosis of the head and neck in children: a study from Sarawak, Malaysia
Source: BMC Infect Dis. 2021 Oct 15;21:1069. doi: 10.1186/s12879-021-06754-9 (PMC8520244; doi:10.1186/s12879-021-06754-9)
Supplement: Supplementary file 3 — Additional file 3: Table S2. Patient, health personnel, and infrastructure characteristics present in Bintulu Hospital (Sarawak, Malaysia) related to the improved diagnosis of melioidosis of the head and neck in children with the use of fine-needle aspiration versus surgical biopsy. [file 12879_2021_6754_MOESM3_ESM.docx]

**Table S2**

**Patient, health personnel, and infrastructure characteristics present in Bintulu Hospital (Sarawak, Malaysia) related to the improved diagnosis of melioidosis of the head and neck in children with the use of fine-needle aspiration versus surgical biopsy**

| No. | Characteristic | Fine-needle aspiration | Surgical biopsy |
| --- | --- | --- | --- |
| 1. | Presentations with features of pyogenic (staphylococcal or streptococcal) infection leading to perceived lack of indication for biopsy | Fine-needle aspiration is a simple and safe procedure with minimal risks leading to lower thresholds for sampling | Unlikely to be performed |
| 2. | Highly variable presentations of melioidosis cervical lymph node disease mimicking malignancy or tuberculosis leading to fears of seeding of tumor or sinus formation with biopsy | Minimal risk | Risk present |
| 3. | Ill patients with disseminated sites of infection | Can still be performed as procedure is conducted at the bedside with minimal sedation | Unlikely to be performed due to operative/anesthesia risks |
| 4. | Patient and parental consent | Preferable to patients/parents as is mostly painless | May be associated with more pain, need for sutures, surgical scars, general anesthesia |
| 5. | Lack of knowledge among health providers on the importance of sampling sites of infection to establish microbiological diagnosis of melioidosis | Allows early sampling with the specific aim of obtaining material for microbiological diagnosis, leading to improved melioidosis diagnosis and patient care. Positive cultures reinforce the usefulness of fine-needle aspiration and leads to positive experiences with the procedure, thus enhancing interest in diagnostic confirmation of melioidosis | Microbiological samples for laboratory diagnosis usually not obtained, or only obtained during therapeutic procedures (i.e., incision and drainage), which are often performed later with negative yield |
| 6. | No pediatric surgery/ otorhinolaryngology service in-house | In-house adult surgeons and/or other trained health personnel are keen to assist by performing fine-needle aspiration as risks are minimal when performed correctly | Rarely performed by adult surgeons and would rather recommend empirical treatment with antibiotics. Nearest center with pediatric surgical service located 300km away making transfer with the sole purpose of biopsy unrealistic |
| 7. | Limited resources including lack of pediatric anesthesia services, operating theater time, funds | Fine-needle aspiration is an inexpensive, easily-performed procedure not requiring general anesthesia | Higher costs and needs general anesthesia |
